# Rapamycin: Killing two birds with one stone

**DOI:** 10.18632/aging.100405

**Published:** 2011-12-11

**Authors:** Amit Khanna, Pankaj Kapahi

**Affiliations:** Buck Institute for Aging Research, Novato CA 94945, USA

Inhibition of TOR signaling leads to extended longevity in both invertebrate and invertebrate species by modulating a number of downstream molecular pathways [1, 2]. Drugs that inhibit the TOR pathway can serve as powerful tools to translate the effects of the TOR pathway on lifespan in simpler invertebrate model systems to more complex systems like mammals. One of the most promising drugs to slow aging is rapamycin, an inhibitor of TOR, which was previously shown to extend lifespan in mice when administered late in life [3]. As aging is one of the biggest risk factors for cancer, one of the outstanding questions is whether drugs that slow aging will also slow age-related increases in cancer incidence. A recent study describes how treatment of mice initiated early in life can not only retard aging but also slow age-related increase in cancer.

Work by Anisimov and colleagues examines the impact on health and lifespan of Rapamycin treated inbred female mice from 2 months of age as compared to control littermates. However, its effects on aging if administered early and intermittently have not been known. Some of the effects include a significant decline in weight gain across the lifespan, a significant increase in regularity of estrous cycle before the onset of old age and more than 20% increase in survival rate along with increase of 10% in median lifespan. Rapamycin targets TOR, a Ser/Thr kinase, the kingpin of a conserved nutrient sensing pathway and causes decline in S6 Kinase (S6K) phosphorylation at nano-molar concentrations (3). It has been earlier reported in an S6k1−/− mouse there is an increase in life span by activation of AMPK and increased resistance against many age related diseases but no effect on age-related increase in cancer incidence [4]. Hence, this study suggests that Rapamycin might have protective effects on age-related cancers through downstream effectors of TOR other than S6K.

What are the possible mechanisms of decrease in age-related cancer incidence in Rapamycin treated mice? As the Rapamycin treated mice not only had a decline in number of tumor bearing mice but also showed an increase in their median lifespan, it is possible that slowing aging prevents the accumulation of age-related cancers. The authors also report an increase in chromosomal aberrations in Rapamycin treated mice as compared to control littermates. Hence protection against DNA damage is unlikely to be the cause of the protective effects of Rapamycin, however it may enhance apoptosis. Earlier higher dose of Rapamycin has been shown to be effective in killing cancerous cells through its apoptotic effects [5, 6]. Furthermore, a recent report shows a cancer cell specific apoptotic effect of high dose of Rapamycin is associated with complete dissociation of Raptor (regulatory associated protein of TOR) from mTORC1 along with decline in phosphorylation of eukaryotic binding protein-1 (4E-BP1) and inhibition of eukaryotic initiation factor 4E (eIF4E) [7]. These observations indicate the possibility that Rapamycin increases lifespan and protection against cancer by decline in phosphorylation of S6K and 4E-BP1 respectively.

One possible reason for the protective effects of Rapamycin on aging in mice may the reduced mortality from cancer. Anismov *et al* found a reduced mortality in mice suffering from tumors [8]. However, the sample size of mice not suffering from tumors was not large to examine whether Rapamycin extends lifespan independently of its effects on cancer. Nevertheless, the study argues that treatment with Rapamycin may help prevent the onset of age-related diseases like cancer and also aging. Furthermore, given slight change in food intake, no change in length of estrous cycle and an illegible change in water intake and body temperature among treated and control groups [8] argues that Rapamycin treatment does not lead to many side effects in this study.

Cancer has been linked with aging since genetic mutations accumulate with age and delayed aging has been associated with lesser incidence of cancers [9]. Activation of TOR is involved in number of aging related diseases including cancer where mutations in upstream factors like PTEN, Ras and PI3K activate TOR signaling [10]. Not all TOR mimetic have been shown to be effective against TOR dependent tumors [11], suggesting Rapamycin ingestion from early age inhibits aging that in turn provides protection against cancer. This study also provides an interesting insight for the possible use of Rapamycin in preventive medicine. Rapamycin could be given to people predisposed to cancer based on genetic and family history and may also be useful to treat cancer patients to prevent relapses and metastasis.
